# Predictive model to differentiate chronic histaminergic angioedema and chronic spontaneous urticaria with angioedema

**DOI:** 10.1016/j.jacig.2024.100389

**Published:** 2024-12-16

**Authors:** Torsten Zuberbier, Ana M. Giménez-Arnau, Marcus Maurer

**Affiliations:** a Institute of Allergology, Charité – Universitätsmedizin Berlin, Corporate Member of Freie Universität Berlin and Humboldt-Universität zu Berlin, Berlin, Germany; b Fraunhofer Institute for Translational Medicine and Pharmacology ITMP, Immunology and Allergology, Berlin, Germany; c Hospital del Mar Research Institute, Department of Dermatology, Barcelona, Spain; d Universitat Pompeu Fabra, Barcelona, Spain

*To the Editors:*

With interest, we read the contribution by Láinez-Nuez et al.^1^ We interpret the data presented in a different way and believe that the results reported do not support changing the nomenclature and differentiating histaminergic angioedema from chronic spontaneous urticaria as an entity. Previous reports^2^ and the data presented show that the similarities between patients with stand-alone angioedema and patients with angioedema with wheals outweigh the differences, and the authors also state this in their conclusion.

Why it is not advisable to change the nomenclature? The current classification of chronic spontaneous urticaria has, for many years, grouped mast cell–mediated stand-alone angioedema as a phenotype of chronic spontaneous urticaria.^3^ In chronic spontaneous urticaria, wheals and angioedema are mast cell–mediated, and it is the degranulation of mast cells in different skin layers that drives the development of wheals versus angioedema. The activation and degranulation of subepidermal mast cells leads to wheals, whereas the activation and degranulation of deep dermal and subcutaneous mast cells leads to angioedema. In some cases, skin lesions after cutaneous mast cell degranulation are hard to identify as either wheals or angioedema because multiple mast cell populations are affected.

Also, the use of a single name for a disease with several subtypes is well established in medicine and helps clinicians and patients to understand that this disease, although it can vary in clinical manifestation, shows shared pathogenic pathways and treatment responses across all subtypes. Patients with chronic spontaneous urticaria with stand-alone wheals, patients with stand-alone angioedema, and patients with wheals and angioedema all show the same beneficial responses to antihistamine treatment and treatment with omalizumab.

Stand-alone angioedema as a phenotype of chronic spontaneous urticaria is paralleled by stand-alone angioedema in chronic inducible urticaria, often depending on the severity of the trigger (eg, in cold urticaria or symptomatic dermographism). Yet it would not be helpful to separate cold-induced whealing and cold-induced angioedema into 2 entities, as they are both cold urticaria.

Coining a new name and changing a well-established classification can lead to confusion for patients and physicians and jeopardize their treatment with drugs that are licensed for the current indication but not for the new one. Patients with the diagnosis “histaminergic angioedema” could be deprived of effective treatment with therapies that are currently licensed for chronic spontaneous urticaria (ie, antihistamines and omalizumab) with detrimental consequences.

## Disclosure statement

Disclosure of potential conflict of interest: T. Zuberbier has received honoraria for lectures from Amgen, AstraZeneca, AbbVie, ALK-Abelló, Almirall, Astellas, Bayer Health Care, Bencard, Berlin Chemie, FAES Farma, HAL Allergie GmbH, Henkel, Kryolan, Leti, L’Oreal, Meda, Menarini, 10.13039/100009947Merck Sharp & Dohme, 10.13039/100004336Novartis, Nuocor, Pfizer, Sanofi, Stallergenes, Takeda, Teva, UCB, and Uriach, as well as fees for industry consulting were from Abivax, Almirall, Bluprint, Celldex, Celltrion, Novartis, and Sanofi; in addition, he declares nonpaid service as a member of the committee Allergic Rhinitis and its Impact on Asthma, a member of the board of the German Society for Allergy and Clinical Immunology, head of the European Centre for Allergy Research Foundation, president of the Global Allergy and Asthma Excellence Network (GA^2^LEN), and a member of the Committee on Allergy Diagnosis and Molecular Allergology of the World Allergy Organization. A. Giménez-Arnau is or was a medical adviser for Uriach Pharma/Neucor, Genentech, Novartis, FAES, GSK, Sanofi-Regeneron, Amgen, 10.13039/100011033Thermo Fisher Scientific, Almirall, Celldex, and LEO Pharma and has received research grants supported by Uriach Pharma, Novartis and Instituto. M. Maurer was a speaker and/or advisor for and/or has received research funding from Allakos, Alexion, Alvotech, Almirall, Amgen, Aquestive, argenX, AstraZeneca, Celldex, Celltrion, Clinuvel, Escient, Evommune, Excellergy, GSK, Incyte, Jasper, Kashiv, Kyowa Kirin, Leo Pharma, Lilly, Menarini, Mitsubishi Tanabe Pharma, Moxie, Noucor, Novartis, Orion Biotechnology, Resoncance Medicine, Sanofi/Regeneron, Santa Ana Bio, Septerna, Servier, Third HarmonicBio, ValenzaBio, Vitalli Bio, Yuhan Corporation, and Zurabio.

## References

[1] Láinez-Nuez A., Salas-Parra G., Juárez-Guerrero A., Picó-Peris A., Baeza M.L. (2024). Predictive model to differentiate chronic histaminergic angioedema and chronic spontaneous urticaria with angioedema. J Allergy Clin Immunol Glob.

[2] Buttgereit T., Vera C., Aulenbacher F., Church M.K., Hawro T., Asero R. (2023). Patients with chronic spontaneous urticaria who have wheals, angioedema, or both, differ demographically, clinically, and in response to treatment—results from CURE. J Allergy Clin Immunol Pract.

[3] Zuberbier T., Abdul Latiff A.H., Abuzakouk M., Aquilina S., Asero R., Baker D. (2022). The international EAACI/GA^2^LEN/EuroGuiDerm/APAAACI guideline for the definition, classification, diagnosis, and management of urticaria. Allergy.

